# Vaccine completion and infectious diseases screening in a cohort of adult refugees following resettlement in the U.S.: 2013–2015

**DOI:** 10.1186/s12879-021-06273-7

**Published:** 2021-06-16

**Authors:** Amir M. Mohareb, Bryan Brown, Kevin S. Ikuta, Emily P. Hyle, Aniyizhai Annamalai

**Affiliations:** 1 Medical Practice Evaluation Center, Massachusetts General Hospital, 16th Floor, 100 Cambridge Street, Boston, MA 02114 USA; 2 Division of Infectious Diseases, Massachusetts General Hospital, Boston, MA USA; 3 Harvard Medical School, Boston, MA USA; 4 Department of Internal Medicine, Yale University School of Medicine, New Haven, CT USA; 5 Division of Infectious Diseases, Veterans Affairs Greater Los Angeles Healthcare System, Los Angeles, CA USA; 6 Division of Infectious Diseases, University of California Los Angeles, Los Angeles, CA USA; 7 Department of Psychiatry, Yale University School of Medicine, New Haven, CT USA

**Keywords:** Refugees, Hepatitis B, Hepatitis C, HIV, MMR, HPV, Immunizations

## Abstract

**Background:**

Refugees are frequently not immune to vaccine-preventable infections. Adherence to consensus guidelines on vaccination and infectious diseases screening among refugees resettling in the U.S. is unknown. We sought to determine rates of vaccine completion and infectious diseases screening in refugees following resettlement.

**Methods:**

We conducted a retrospective cohort study of refugees resettling in a region in the U.S. using medical data from June 2013–April 2015. We determined the proportion of vaccine-eligible refugees vaccinated with measles-mumps-rubella (MMR), hepatitis A/B, tetanus, diphtheria, and acellular pertussis (Tdap), and human papillomavirus (HPV) following resettlement. We also determined the proportion of refugees who completed HIV and hepatitis C (HCV) screening.

**Results:**

One hundred and eleven subjects were included, primarily from Iraq (53%), Afghanistan (19%), and Eritrea (11%). Of the 84 subjects who were vaccine-eligible, 78 (93%) initiated and 42 (50%) completed vaccinations within one year of resettlement. Odds of completing vaccination were higher for men (OR: 2.38; 95%CI:1.02–5.71) and for subjects with English proficiency (OR: 3.70; 95%CI:1.04–17.49). Of the 78 subjects (70%) completing HIV screening, two (3%) were diagnosed with HIV. Nearly all subjects completed screening for HCV, and one had active infection.

**Conclusion:**

While most refugees initiate vaccinations, only 50% completed vaccinations and 70% completed HIV screening within 1 year of resettlement. There is a need to emphasize vaccine completion and HIV screening in refugee patients following resettlement.

## Background

There has been an unprecedented rise in vaccine-preventable infections around the world, including in immigrant and refugee communities [1, 2]. The number of refugees being resettled in the U.S. was severely limited between 2017 and 2020, but it is expected to rise in the coming years due to anticipated changes in federal policy on refugee resettlement. Unlike most immigrant groups in the U.S., U.S.-bound refugees are not required to receive vaccinations prior to arrival [3]. Prior studies in refugees have demonstrated low rates of baseline immunity to vaccine-preventable infections, including measles, mumps, rubella (MMR), and viral hepatitis [4–9]. To address this, the U.S. Centers for Disease Control and Prevention (CDC) and the U.S. Department of State developed a pre-departure vaccination program for U.S.-bound refugees, which has been growing in implementation since 2013 [10]. However, gaps in immunity may still persist, particularly in refugees resettled prior to or in the early years of this program. Although guidance from the CDC recommends vaccinating eligible refugees following resettlement [3], the proportion of refugees who complete post-resettlement vaccination, especially of multidose vaccines, is unknown.

Screening practices for HIV and hepatitis C virus (HCV) among refugees in the post-resettlement period are also unknown. Mandatory pre-departure HIV screening has not been conducted in U.S.-bound refugees since 2010 [11, 12]. Therefore, HIV screening is recommended for all refugees following resettlement [12], but these recommendations may not be consistently followed [13, 14]. Our objective was to determine the proportion of refugees who complete post-resettlement vaccination and HIV and HCV screening.

## Methods

### Population

We conducted a retrospective cohort study of adult refugees resettling in a district in Connecticut, U.S. All refugees resettling in this region underwent screening in a dedicated clinic and continued to receive follow-up care in the same primary care network. We considered adult refugees (age at least 18 years-old at the time of resettlement) eligible for inclusion if they had 1 year of follow-up clinical data after completing a medical screening exam, which included serologic testing of immunity by immunoglobulin G (IgG) for measles, mumps, rubella (MMR), hepatitis A, hepatitis B, and varicella. Immunity testing for tetanus, diphtheria, or pertussis (Tdap) was not available. We considered subjects eligible for one of these vaccines if they did not have a positive IgG or evidence of overseas documentation of vaccination. We considered all males and females 26 years-old and younger to be eligible for three doses of the human papillomavirus (HPV) vaccine [15, 16]. We ascertained the proportion of eligible refugees who initiated HPV vaccine series because none were previously vaccinated prior to resettlement. We also determined the proportion of refugees who completed screening for HIV by a fourth generation antibody-antigen test and HCV by IgG antibody testing.

### Data collection

We collected data from the medical record on subjects’ age, sex, country of origin, and English proficiency, defined as not requiring a medical interpreter during clinical visits. We defined country of origin as the country of primary residence immediately prior to resettlement. We captured vaccinations and HIV/HCV screening information from the time of initial evaluation until 1 year following resettlement for each subject. We used this timeframe because it encompasses the period during which federal health insurance (i.e., Refugee Medicaid Assistance) is available in the U.S. for this patient population [17]; therefore, refugees in this cohort should have no lapses in health insurance coverage during the study, which might otherwise affect vaccine completion. We included all refugees who resettled in this region from June 1, 2013, to April 30, 2014, and collected data through April 30, 2015. All subjects were confirmed to remain in the same primary care network for 1 year following resettlement. These dates of inclusion were chosen because after April 2014, refugees newly resettling in this region were not consistently retained in the same primary care network, so follow-up vaccine completion would not be reliably ascertained.

### Statistical analysis

We analyzed the proportion of subjects who were immune and the proportion of vaccine-eligible subjects who initiated and completed MMR, hepatitis A/B, Tdap, and HPV vaccinations within 1 year after resettlement. Varicella vaccine was not readily available for administration in the clinic, so we reported baseline varicella immunity only. We also analyzed MMR and hepatitis A/B in aggregate by the following definition: those non-immune to any of these diseases were considered non-immune to the aggregate and were vaccine-eligible; among those who were vaccine-eligible, only those completing all necessary vaccines so that they were fully immune were considered successfully vaccinated. We evaluated predictors of complete vaccination at 1 year post-resettlement among those who were vaccine-eligible by univariable analysis. We defined statistical significance as two-tailed *p* < 0.05, and we used R version 3.5.1 for statistical computation. The sample size of this cohort did not allow for a stable multivariable analysis of our characteristics of interest: age, sex, country of origin, and English language proficiency. This study was approved by the institutional review boards of participating institutions.

## Results

A total of 111 subjects met inclusion criteria for this study (Table 1). The mean age was 34 years (range: 19–76 years), and 41% were female. Countries of origin included Iraq (53%), Afghanistan (19%), Eritrea (11%), and Ethiopia (5%); the remainder (13%) were from other countries of origin (i.e., Sudan, Syria, the Democratic Republic of Congo, and Colombia). The median time between resettlement in the U.S. and initial assessment in the refugee clinic was 34 days (interquartile range: 15–44 days).

**Table 1:** Baseline Characteristics of Adult Refugees Resettling in a Connecticut district, 2013–2015

Variable	Value
Total participants, n	111
Age, median (IQR), years	30 (18–76)
Sex, n (%) female	45 (41%)
Country of Origin, n (%)	
Iraq	59 (53%)
Afghanistan	20 (19%)
Eritrea	12 (11%)
Ethiopia	6 (5%)
Other^a^	14 (13%)
Time between resettlement and screening, median (IQR), days	34 (15–44)
English proficiency, n (%)	20 (18%)
Resettled simultaneously with family (> 1 person), number (%)	85 (77%)

Note: *IQR* Interquartile range

^a^ Other countries include Sudan, Syria, the Democratic Republic of Congo, and Colombia

**Fig. 1:**
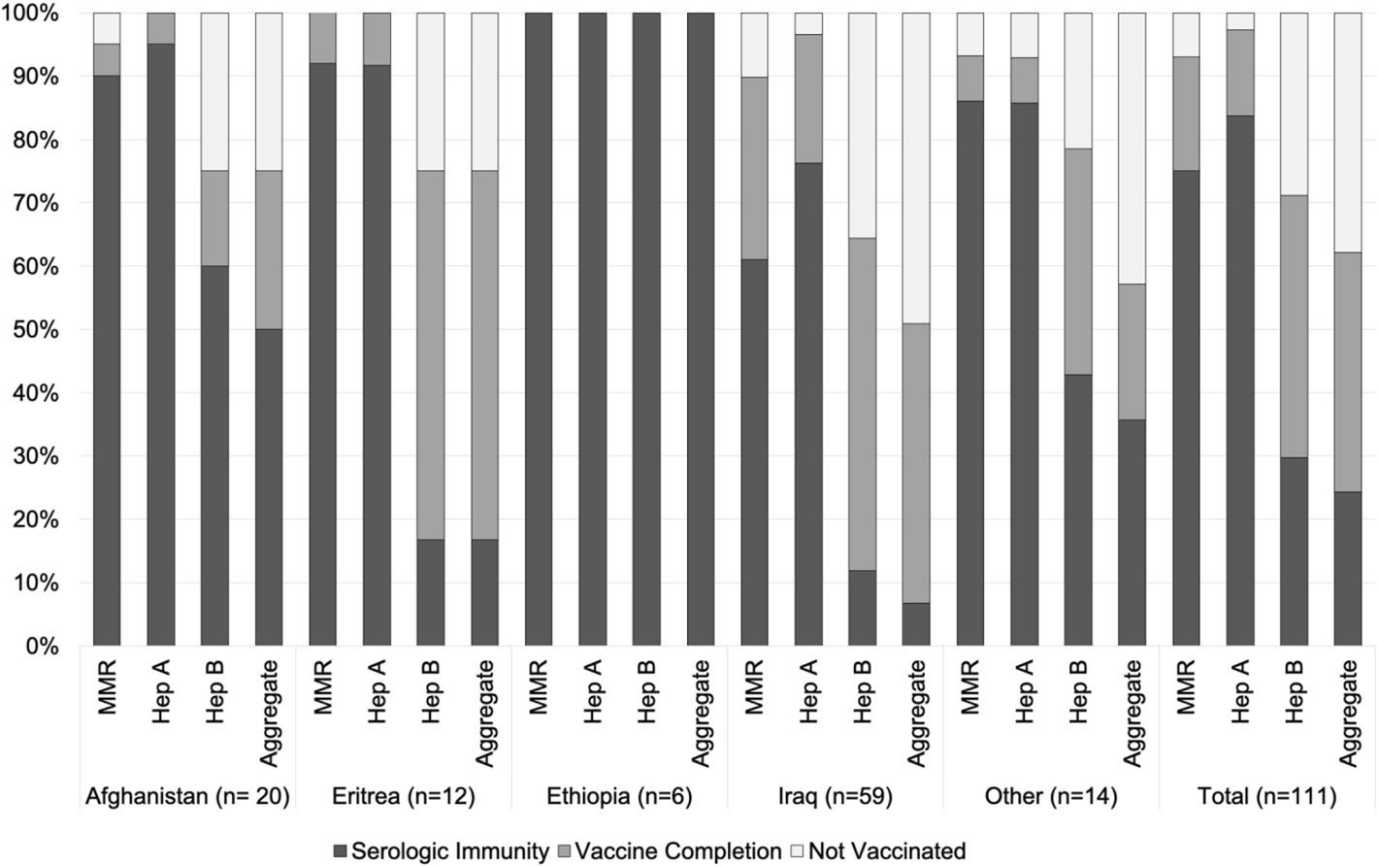
Serologic immunity and vaccine completion for multi-dose vaccine series up to one year after resettlement in a cohort of refugees in Connecticut, 2013–2015. Note: MMR = measles, mumps, rubella; Hep A = hepatitis A; Hep B = hepatitis B

Aggregate baseline immunity to all of measles, mumps, rubella, hepatitis A, and hepatitis B was present in 27 (24%) subjects (Table 2). Of the remaining 84 subjects who were vaccine-eligible for MMR, hepatitis A, or hepatitis B, 42 (50%) were fully vaccinated within 1 year of resettlement date. Serologic immunity and vaccine completion varied by country of origin (Fig. 1). Twenty-eight subjects (25%) were non-immune to any of measles, mumps, or rubella. Of these 28 subjects, 20 (71%) completed the two-dose MMR vaccine series, 6 (21%) received one dose of the series, and 2 (8%) did not initiate the series within 1 year of resettlement. Seventy-eight subjects (70%) were non-immune to hepatitis B. Of these, 46 (59%) completed the three-dose vaccine series within 1 year, 26 (33%) had initiated but not completed the series, and 6 (8%) did not initiate the series within 1 year of resettlement. Nearly all subjects (96%) were vaccinated with Tdap after resettlement. Baseline immunity to varicella was present in 90% of subjects. Thirty-four subjects met criteria for the HPV vaccine series (i.e., age below 26 years), of whom 5 (15%) received at least one dose during the study period.

**Table 2:** Baseline Vaccine-Eligibility, Initiation, and Completion of Recommended Vaccinations Among Adult Refugees in a Connecticut district, 2013–2015

	Baseline Immunity Among All 111 Subjects at the Time of Resettlement n (%)		Vaccinations One Year after Resettlement Among Vaccine-Eligible Subjects n (%)	
Vaccine	Immune	Non-immune (Vaccine-Eligible)	Initiated	Completed
Aggregate^a^	27 (24%)	84 (76%)	78 (93%)	42 (50%)
MMR (2 doses)	83 (75%)	28 (25%)	26 (93%)	20 (71%)
Hep A (2 doses)	93 (84%)	18 (16%)	16 (89%)	15 (83%)
Hep B (3 doses)	33 (30%)	78 (70%)	72 (92%)	46 (59%)
Tdap (1 dose)	N/A	111 (100%)	106 (96%)	106 (96%)
HPV (3 doses)^b^	N/A	34 (31%)	5 (15%)	1 (20%)
Varicella	100 (90%)	11 (10%)	N/A	N/A

Note: *MMR* Measles, mumps, rubella, *Hep A* Hepatitis A, *Hep B* Hepatitis B, *Tdap* Tentanus, diphtheria, acellular pertussis, *HPV* Human papillomavirus. Baseline immunity determined by laboratory testing for immunoglobulin G or documentation of completed overseas vaccination

^a^ Aggregate of measles, mumps, rubella, hepatitis A/B; we considered subjects to be vaccine-eligible if they required any of these vaccines

^b^ HPV vaccine eligibility was assumed for all subjects < 26 years-old. No subject had documentation of prior HPV vaccination

Among those who were vaccine-eligible, older age was associated with a lower odds of vaccine completion (for each year increase in age, OR: 0.96, 95%CI: 0.93–0.99). In particular, subjects older than 50 years-old had a much lower odds of vaccine completion compared to those younger than 50 years-old (OR 0.12, 95%CI: 0.02–0.47). A higher odds of vaccine completion was noted in subjects who were male (OR: 2.38, 95%CI: 1.02–5.71) and English speakers (OR: 3.70, 95%CI: 1.04–17.49). Country of origin was not associated with a difference in vaccine completion (Table 3).

**Table 3:** Factors Associated with Vaccine Completion Among Adult Refugees in a Connecticut district, 2013–2015

	Odds Ratio (95% CI) of Vaccine Completion^**a**^ (***n*** = 84)
Age (per year)	0.96 (0.93–0.99)*
Age (binary)	
Age < 50	Reference
Age ≥ 50	0.12 (0.02–0.47)*
Sex	
Female	Reference
Male	2.38 (1.02–5.71)*
English proficiency	
No	Reference
Yes	3.70 (1.04–17.49)*
Country of Origin	
Iraq	Reference
Afghanistan	0.42 (0.12–1.25)
Eritrea	2.54 (0.72–10.38)
Ethiopia	0.25 (0.01–1.70)
Other^b^	0.95 (0.28–3.08)

^a^ Tdap, MMR, hepatitis A/B

^b^ Comprised of Sudan, Syria, the Democratic Republic of Congo, and Colombia

* *p* < 0.05

Of the 78 subjects (70% of the cohort) completing HIV screening, two (3%) were diagnosed with HIV after confirmation with HIV RNA testing. Both were successfully linked to care at a local HIV clinic. One hundred and ten subjects (99% of the entire cohort) completed screening for HCV; two subjects (2%) were positive for HCV Ab, of whom one had detectable HCV RNA.

## Discussion

Among 111 resettled refugees, 84 (76%) were eligible for at least one of the following vaccines: MMR, hepatitis A, or hepatitis B. Although 93% of vaccine-eligible subjects initiated vaccination(s), only 50% completed vaccinations within 1 year of resettlement. This demonstrates that refugees who resettled in the U.S. during this time period (2013–2014) remain vulnerable to vaccine-preventable infections long after resettlement.

A plausible reason for incomplete immunization is patient acceptability to return to clinic for multi-dose vaccine series. This may be why the three-dose series for hepatitis B had a lower completion rate (59%) than hepatitis A (83%) or MMR (71%), both of which are two-dose series, and Tdap (96%), a one-dose series. One factor that may improve these rates in the U.S. is implementation of the CDC Overseas Vaccination Program for U.S.-bound Refugees, a program which initiates age-appropriate vaccinations at international asylum sites in the months prior to departure to the U.S. At the time of our study, this program was beginning to be implemented in six countries but has since expanded to all sites processing U.S.-bound refugees. Documentation of overseas vaccinations at these sites is available to health providers in the U.S. through the CDC Electronic Disease Notification system [18].

The refugee health assessment is an important opportunity to ensure MMR immunity among refugee patients [1]. In this cohort, one in four subjects required MMR vaccination after resettlement. A prior study found a lower rate of MMR-eligible refugees (10%) from a sampling of asylum countries in East Africa and Southeast Asia [5]. That study found that 4% of MMR-eligible refugees did not receive a dose of the MMR vaccine during their initial encounter for the refugee health assessment, but they were unable to determine if vaccination was administered in subsequent encounters. Our study fills this research gap by looking at the post-resettlement period for a duration of 1 year. In our cohort, 8% of MMR-eligible subjects did not receive any doses of MMR vaccine within 1 year of resettlement, while 21% had received only one of the two-dose MMR vaccine series. This represents missed opportunities for the prevention of disease transmission, particularly in an era of frequent measles and mumps outbreaks.

Refugees in the U.S. frequently resettle from countries that lack nationwide hepatitis B vaccine programs, even though global hepatitis B vaccination coverage is improving [3]. Thirty percent of our cohort had baseline immunity against hepatitis B, which is comparable to a prior multi-center study of refugees [6]. We found that among those eligible for vaccination, only 59% completed the three-dose hepatitis B vaccine series within 1 year of arrival. This compares favorably to the general U.S. adult population which reports 25% vaccine coverage for hepatitis B [19]. However, this value is still too low given that individuals from hepatitis B-endemic countries remain at risk of incident infection even after resettlement in non-endemic countries [20].

Ten percent of subjects in this cohort were non-immune to varicella at the time of resettlement, which is consistent with previously published observations [7]. Varicella seroprevalence varies by age in different regions in the world; other studies have noted high varicella susceptibility in persons originating from tropical climates [21]. Varicella vaccination is not routinely available in many adult clinics (including ours at the time of this study) and therefore can be more challenging to provide for eligible adult patients. However, vaccination should be a priority since complications of varicella are more likely when primary infection occurs in adulthood [22].

Our study also evaluated the HPV vaccine series in eligible subjects. We found that among both men and women in our study cohort below the age of 26 years, only 15% had initiated the HPV vaccine series within 1 year of resettlement. Prior studies have noted marked disparities in HPV vaccination and cervical cancer screening among foreign-born persons in North America [23, 24]. A study of female Vietnamese-Americans found comparable rates of HPV vaccination and specifically noted an increased odds of vaccination among those who correctly recognized that HPV infection was not curable with medications [25]. This suggests room for improved counseling and education regarding the nature of HPV infection as a means to increase vaccination rates among foreign-born persons.

An important finding of this study was that only 70% of the cohort completed HIV screening, whereas 99% completed HCV screening. These data suggest a persistent stigma regarding HIV among refugee patients or a lack of perceived risk, despite an increased prevalence of risk factors for HIV and HCV infection among refugees, including exploitation during the migration process, exposure to sexual violence, and unregulated healthcare procedures [11]. HIV prevalence was 3% among those who underwent screening, which is comparable to a prior study of refugees resettling in Washington D.C. and higher than the general U.S. population [4]. Since 2010, refugees being resettled in the U.S. have not been required to undergo pre-departure HIV testing; HIV testing and counseling are therefore a crucial intervention in the refugee health assessment after arrival [11]. Our study reinforces the need to screen all refugees for both HIV and HCV in the current era of safe and effective therapies for both diseases to reduce the risk of long-term complications and transmission.

In a univariable analysis of predictors of vaccine completion among the vaccine-eligible, older age was associated with a lower odds of completing vaccination, particularly for those subjects greater than 50 years old. This may be related to decreased health literacy among older refugees or competing health issues for patients with more chronic conditions during follow-up visits. Subjects who were male and English speakers had greater odds of completing vaccination. This may be related to disparities in health literacy or challenges in navigating the healthcare system among refugees who are female and/ or non-English speakers [26, 27]. We do not believe that access to care or health insurance was a key determinant of these differences since all refugees in this cohort had insurance for the duration of this study and were seen in the same primary care network for other health needs.

These results should be interpreted within the limitations of study design. The seroprevalence estimates in this study may not reflect refugees who arrived in the U.S. after 2014, given the expansion of the Overseas Vaccination Program for U.S.-bound refugees and federal changes to refugee policies [10]. These data are from a single region in the U.S., serving refugees primarily from Iraq, Afghanistan, and East Africa. As compared to the rest of the state of Connecticut, and the remainder of the U.S., our study population under-sampled refugees originating from Southeast Asia, South Asia, Central/ South America, and Central Africa [28]. The sample size of this study population limited the precision of the point estimates and precluded a multivariable analysis. Therefore, the associations we found with vaccine completion may be subject to confounding. Although we could not capture vaccine administration in other healthcare settings, this was likely a rare occurrence because patients returned to the same primary care network for the rest of their healthcare needs. Further studies are needed to examine the reasons for incomplete vaccination and to develop approaches to improve vaccination among adult refugees. Reasons for incomplete vaccination in refugees, and effective approaches to improve vaccine completion, may be relevant to the general U.S. population more broadly [19].

## Conclusions

In conclusion, almost all refugees eligible for vaccination with MMR, hepatitis A, and/ or hepatitis B initiated the recommended vaccination(s), but only 50% completed immunization within 1 year of their resettlement. Among those who completed HIV and HCV screening, 3% of refugees were newly diagnosed with HIV and 1% with chronic HCV. Refugee health providers must emphasize vaccinations and screening for chronic, treatable infections for their patients.

## Data Availability

The datasets used and/or analysed during the current study are available from the corresponding author on reasonable request.

## Abbreviations

MMR: Measles, mumps, and rubella
CDC: Centers for Disease Control and Prevention
HCV: Hepatitis C virus
IgG: Immunoglobulin G
Tdap: Tetanus, diphtheria, acellular pertussis
HPV: Human papillomavirus
